# Is More Better? Insights on Consumers’ Preferences for Nutritional Information on Wine Labelling

**DOI:** 10.3390/nu10111667

**Published:** 2018-11-04

**Authors:** Riccardo Vecchio, Azzurra Annunziata, Angela Mariani

**Affiliations:** 1 Department of Agricultural Sciences, University of Naples Federico II, 100 Via Università, 80055 Portici, Italy; riccardo.vecchio@unina.it; 2 Department of Economics and Legal Studies, University of Naples Parthenope, 13 Via Parisi, 80133 Naples, Italy; mariani@uniparthenope.it

**Keywords:** nutritional information, back label, willingness-to-pay (WTP), experimental auctions

## Abstract

Background: Nowadays there is a strong debate on the need to introduce mandatory nutritional information on alcoholic beverages labels, and particularly on wine, as a tool to promote more health-conscious drinking patterns in society. In 2018, the European alcoholic beverages industry presented a self-regulatory proposal, now under assessment by the European Commission. The most critical issue is how to convey nutritional information to consumers, as producers should decide to apply information on label or off-label. Method: The current study measured, through a non-hypothetical, incentive compatible artefactual field experiment, Italian wine consumers (*N* = 103) preferences for four different formats of wine nutritional labelling, namely: back label with the indication of kcal for glass of wine, with the nutritional panel referred to 100 mL, without nutritional information (but with a link to an external website) and with the indication of key nutrients for glass of wine. Results: Findings reveal that respondents preferred the nutritional panel on the back label, assigning the lowest preference to the less informative wine label (only with a website recall). Furthermore, results show a low level of respondents’ knowledge of wine nutritional properties. Conclusion: Findings, while limited in terms of sample representativeness, seem to support the European Consumer Organisation and the European Alcohol Policy Alliance objection to an off-line label and the advocacy for a traditional and complete on label nutritional information on wine.

## 1. Introduction

The harmful use of alcohol has a severe impact on health and well-being of individuals and imposes significant social and economic costs on society [1]. Despite this, alcoholic beverages are subject in many respects to milder forms of regulation not only compared to other psychoactive substances but also to foods [2]. Labelling requirements is one of the aspects for which alcoholic beverages have benefited from special treatment.

Nowadays, in several countries, there is an ongoing debate on the need and usefulness to introduce mandatory nutritional information and ingredients list on alcohol beverage labels as a tool to promote more health-conscious drinking patterns in society [3,4]. Following the World Health Organisation’s European Action Plan to reduce the harmful use of alcohol (2012–2020), the alcoholic beverages’ labelling should be similar to other foods to ensure that consumers have access to complete information on the content and composition of the product for the protection of both their health and interests [5]. In the European Union, after a fierce debate on the extension of Reg. 1169/2011 to wine and other beverages with an alcohol content of more than 1.2% by volume, on March 2017, the European Commission (EC) presented its report concluding that nutrition labelling requirements should be extended to alcoholic beverages [6,7]. The EC has invited the alcoholic beverages industry to present a self-regulatory proposal covering all involved sectors (wine, beer and spirits). On March 2018, the EU alcoholic beverages industry presented a “joint” self-regulatory proposal on nutrition labelling and ingredients listing to the EC. The joint proposal outlines the general principles of the labelling schemes shared by the alcoholic beverages industry and is accompanied by four sector-specific implementation plans (wine and aromatised wine, spirit drinks, beer, and cider and fruit wine). Focusing on the wine and aromatised wine sector, producers are committed, starting from March 2021, to provide consumers the nutrition declaration and the ingredients list [8]. However, producers keep the possibility to: limit the information to energy value; add the declaration per portion; use symbols; and base the data provided on generally established and accepted average values, given for certain specific wines to reflect the very changing nature of wine (depending on weather conditions and years of production). Furthermore, producers will be able to decide individually how to convey the information at best: on label or off-label, by using online mechanisms accessible from the label (introducing a web-link, or a QR code, an icon providing consumers easy and direct access to the information on line). These off-label tools will be developed at European Union, national, regional, organisation and/or company levels. At the European level, the Wine in Moderation website (https://www.wineinmoderation.eu) will be one of these default solutions for operators [9]. According with this proposal, the use of off-label communication tools, as an alternative to the use of the label, considers both the specificities of the wine sector (e.g., large number of small and medium enterprises (SME)) and the new trends of wine buying behaviour (on-line shopping and increase of information sourcing on the Internet). Therefore, the aim is developing tools that are manageable by SME and, useful and easy to use by final consumers [10].

Currently, this proposal is under assessment by the EC. Should the EC consider this self-regulatory approach as unsatisfactory, it would launch an impact assessment to review further available options. Up to now, the European consumer Organisation (BEUC) points out that, allowing the application of an off-label mechanism, the main function of labelling as direct shopping aid would be lost, as well as its usefulness in aiding consumers to easily compare between several bottles on the shelf [11]. Overall, BEUC expresses concern about recognising this flexibility to alcohol producers as it could create inequities on the market and could prompt other actors in the food and drink industry to demand similar conditions for their products. In addition, the European Alcohol Policy Alliance [12] underlines that the off-label mechanism is not an option as there will always be a segment of European citizens that do not use Internet on a daily base. Furthermore, a relevant share of Europeans does not own a smart phone, which precludes the use of tools such as QR code; in addition, scanning a QR code is time consuming.

In this context, it is crucial to examine consumers’ needs and preferences for nutritional information, delivered both on label and off-label, to provide useful indications for policy makers in the selection of the approach that should be implemented. The current study analysed consumers’ preferences for different format of wine nutritional labelling, through a non-hypothetical, incentive compatible experiment.

## 2. Literature Background and Research Hypothesis

Even though front and back labels are important sources of information for wine consumers [13], the literature offers a limited amount of research that has analysed the issue of individuals’ interest in nutritional information and specific preferences for different format of nutritional labels on wine. Among the available studies, some reveal the strong interest expressed by consumers in nutritional and health information on wine labels [14,15,16,17,18,19] even if considerable differences exist between countries and with reference to the diverse formats to convey the information. In this regard, Grunert and colleagues (2018) found a stronger interest towards information sources alternative to the product label, as public and health-related websites, while also revealing the need to receive information in-store [19]. As for the impact of nutritional information on consumers’ decision, a Commission-mandated study shows that after having been informed about the energy content of alcoholic drinks consumers motivated by health goals declared their intention to reduce their alcohol consumption [20]. Other studies, instead, highlight that the inclusion of nutritional information may influence wine’s healthiness perception and induce unintended consequences [18,21,22,23]. In particular, the availability of serving facts information on wine could decrease calorie and carbohydrate evaluations and increase consumption intentions.

Based on the incontinence of existing research, it seems necessary to offer further evidence on consumers’ needs and preferences for nutritional information on wine label, especially in European countries, due to the importance that the topic is assuming. Furthermore, as all previously mentioned studies are based on stated preferences (as surveys), it could be useful to deepen the analysis through data from non-hypothetical research. In the light of this background, the current study focuses on the following research questions: (1) Are consumers interested in nutritional information on wine? (2) What nutritional label format do consumers prefer on wine? (3) Which are the determinants that affect consumers’ preferences for different wine nutritional labels?

According to the existing literature on food nutritional labelling [24,25,26], there are several stages influencing consumers’ interest in, and use of nutritional information: from exposure to perception, understanding and inference-making to final choice. Each step of this framework is influenced by a number of factors, such as general interest in nutrition, knowledge about nutrition issues and consumer socio-demographics [24,27,28]. Other research suggests that product involvement also acts as motivational determinant in influencing consumer search for nutritional information [16,17,19]. In addition, label format is expected to have an impact on perception, whereas it is an essential aspect in determining consumers’ liking of the label. Consumers may be influenced by one format rather than another because they find it easy to understand and useful, or just because they like the symbols and colours used [29]. This latter aspect is particularly apparent for a product like wine, for which the label format is considered a very important attribute in influencing consumer choices [30,31,32].

## 3. Materials and Methods

### 3.1. Experimental Procedure and Stimuli

To achieve the research objectives, we performed an Artefactual Field Experiment (AFE), recruiting regular buyers of wine. Specifically, the AFE involved individuals, taking part in incentive-compatible, non-hypothetical experimental auctions, random *n*th price auction. The random nth price auction combines elements of two the demand-revealing mechanisms: Vickrey auction and Becker–DeGroot–Marschak (BDM) mechanism (for a complete overview of the mechanism, see Shogren et al. (2001) [33]). This mechanism was selected as experimental auctions have an increasing relevant role in consumer studies [34], estimating individual preferences-measured trough willingness-to-pay (WTP)—for wine attributes [30,35,36,37], as well as for label formats [38].

Following similar studies, the sample size was set at 100 participants (e.g., [39,40]). Participants were recruited informing they would participate in a marketing research survey at the university campus that would last approximately half an hour. Subjects had to meet certain criteria, including: (i) be over 21 years old; (ii) buy a bottle of wine at least once a month; and (iii) consume wine at least once a week.

A within-subject experimental design was performed, organising 10 sessions with 10 ± 2 participants each. A total of 103 consumers were involved. The sessions lasted about half an hour, were spread over five weekdays and involved six consecutive stages: (1) individuals signed a consent form and a form committing them to buy the product if they won; receiving then a compensation for the time devoted to the experiment (€10); (2) participants were seated in individual booths and were strongly requested to avoid any form of communication; (3) a researcher explained the auction mechanism in detail, providing examples and running a training auction with four chocolate bars; (4) participants were handed out the four wine bottles, one by one, and were asked to express a sealed bid for each product; (5) post-auction survey was performed; and (6) a product and a number were randomly drawn (market price); thus, all individuals who offered more than the market price paid and purchased the auctioned wine.

**Figure 1:**
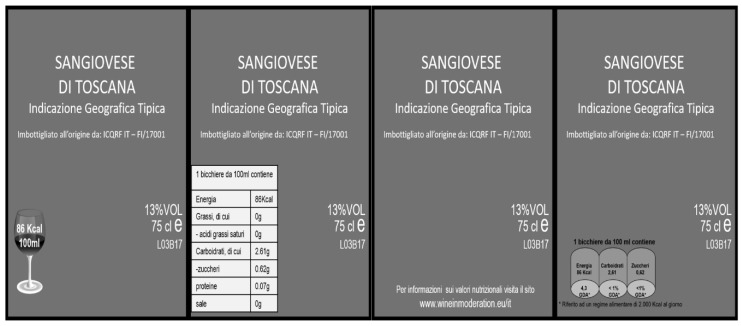
Stimuli applied in the AFE.

No deceptive practice was applied in the study. The wine label was designed by a professional graphic designer, including all the compulsory information required by the national legislation. The four bottles differed only for the nutritional information displayed on the back label (see Figure 1). Specifically: the first back label had the indication of kcal only for glass of wine (Label A); the second label included the nutritional panel referred to 100 mL (Label B); the third was without nutritional information but with a link to an external website—www.wineinmoderation.eu.it (Label C); and the fourth included the Guideline Daily Amount (GDA) labelling with the indication of key nutrients for glass of wine (Label D). To reach a high degree of realism, a widely popular wine in the Italian market was selected (i.e., Sangiovese), in the most commercially available vintage at the time of the experiment (2016). Therefore, the four products, beyond nutritional info, carried exactly the same features (bottle shape, colour and weight, cork type, alcohol content, origin, etc.); were completely unbranded; and no tasting was performed during the experiment. Presentation order of products was randomised across sessions according to a Williams’ Latin square design, balanced for order and first-order carry-over effects.

### 3.2. Questionnaire Design and Measurements Applied

Additional data on the drivers of consumer WTP was collected through a post-auction online questionnaire administrated via on-line platform. The questions aimed to map the participant’s profile with reference to the following main issues: health interest in food choices; labelling attention and use; wine consumption habits and wine involvement; wine nutritional knowledge; awareness of linkages between wine and health; wine nutritional information search and wants; and socio-demographic characteristics.

Health interest in food choices was measured evaluating respondents’ agreement to five statements on a five-point scale (ranging from 1 = strongly disagree to and 5 = strongly agree) adapted from the General Health Interest scale [41].

Labelling attention and use was assessed using three questions adapted from previous researches [42,43].

Wine consumption habits were measured asking participants to indicate frequencies of consumption, place of consumption, shopping channel use and average price payed for the latest bought bottle.

Product involvement was detected applying four statements from a validated wine involvement scale, adapted from Mittal and Lee (1989) [44], widely used in wine consumer research [45,46].

To assess wine nutritional knowledge, two measurements were applied: self-reported knowledge and objective knowledge. Self-reported nutritional knowledge construct was assessed by asking participants to indicate their degree of knowledge on wine nutritional properties (five-point scale, ranging from 1 = not at all to 5 = very much). Objective nutritional knowledge was measured by asking participants to indicate the kcal and carbs content of different types of wine (white and dessert wine) through a multiple-choice question. We also asked to indicate kcal and carbs content of whole bread (as control) and we used the mean value of these answers to build an objective nutritional knowledge.

Awareness of linkages between wine and health was measured using five statements used in previous research [23,47] related to: the potential side effects as well as beneficial effects of wine; the link between wine consumption and obesity; and the perception of wine as alcoholic beverage healthier than others.

Wine nutritional information search was measured firstly by asking participants to indicate how often they search nutritional information on wine, on a five-point scale from 1 = never to 5 = always. Then, according with Grunert and colleagues (2018) [19], participants had to indicate how often they use several information sources to access this information (advertising on TV or magazines; producers’ websites or Facebook page; public information we site; banner or poster in store; QR code; and applications for smartphone/smart watch). In the same way, we asked participants to indicate their degree of interest in receiving information on wine nutritional properties in general and using the same list of information sources. In addition, we asked participants to indicate their degree of interest in receiving information on wine ingredient list on label. Finally, main socio-demographic variables were detected.

For all scales applied in the study, Cronbach alphas provided satisfactory scale reliability coefficients (construct mean value, standard deviation and Cronbach alphas are reported in Appendix).

Collected data were first submitted to descriptive univariate analysis to provide an overview of consumers’ characteristics. The drivers of preferences (measured through WTP) were estimated applying a seemingly unrelated regression (SUR). The SUR is a multivariate linear regression model that allows the estimation of a system of equations; specifically, four equations were estimated, one for each back label format. All data analyses were carried out using STATA 15.

## 4. Results

### 4.1. Descriptive Statistics

A total of 103 wine consumers were recruited in the metropolitan area of Naples (Southern Italy). Considering the socio-demographic profile, 51% of participants are women, the average age of participants is 29.12 (S.D. 7.12). With reference to the education, 33% have obtained an undergraduate degree, while 27% have a graduate degree. Overall, 45.6% belong to the middle-income range (€30,000–50,000 per year). With reference to health interest in food choices, Table 1 shows that participants state to be particularly interested in health aspects of food, as in more than 60% of cases individuals regularly follow a balanced diet, while 54% of respondents consider very important to follow a low in fat diet. However, 33% of participants state to not worry much about the healthiness of food. Considering the attention and use of nutritional labels, 45% totally agree with the statement “I read the nutritional information on the label when I buy food/beverages”, and 42% totally agree with the statement “I use nutritional information to compare products”.

Taking into account wine consumption variables, Table 2 shows that in most cases participants stated consuming wine more than once a week (37%), while 15% once a day. In 57% of cases, consumption takes place mainly at home or at friend’s house and the average cost for a bottle of wine consumed at home is €7.31. Supermarket is the main place of wine purchase (46%), while only 4% buy primarily online.

Table 2 also reveals that the degree of self-reported knowledge of wine nutritional properties is low for 36% of respondents. This is also confirmed by data related to objective wine nutritional knowledge, revealing a low degree of respondents’ knowledge of wine kcal and carbs content.

Considering the variables related to wine involvement, Table 3 shows that participants quite agree with the statement “I don’t need a special occasion to drink wine” (*M* = 3.6), as well as with the statement “I have a strong interest in wine” (*M* = 3.2). Respondents also like giving wine as a gift (*M* = 3.4). 

With reference to knowledge of wine and health linkages, analysing the mean values reported in Table 3, it is interesting to highlight that most of respondents agree with the statement that excessive consumption of wine could affect their health (*M* = 4.1). Moreover, wine holds a better perception in terms of health properties compared to other alcoholic beverages (*M* = 3.8). Furthermore, participants also agree with the statement that moderate consumption of wine can have beneficial effects on health (*M* = 3.6). However, less awareness is witnessed with respect to the link between wine consumption and obesity, and lower agreement is attached to the statement “A moderate wine consumption could help me to prevent some diseases”.

### 4.2. Consumers Search and Wants of Wine Nutritional Information

**Figure 2:**
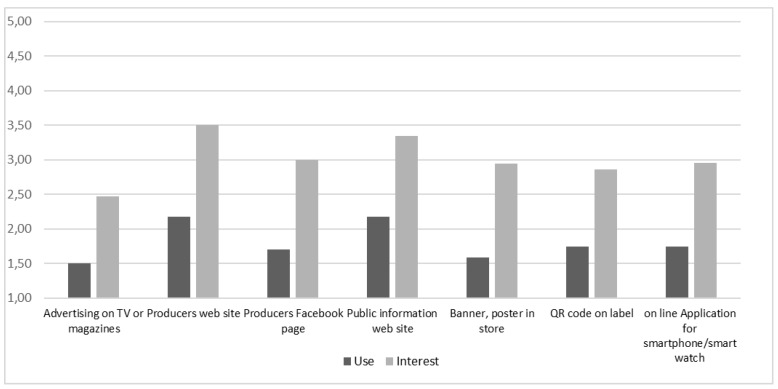
Participants’ use of and interest towards alternative sources for wine nutritional information. Note: Use was measured on a scale from 1 = never to 5 = always. Interest was measured on a scale ranging from 1 = not at all to 5 = very interested.

Results in Table 2 show that in most cases the participants stated not having looked for wine nutritional information (24% never, 31% rarely) while only 8% stated having always looked for this information. Considering the sources used to search for wine nutritional information, as shown in Figure 2, the average level of use for all seven sources proposed is very low; however, producers’ websites (*M* = 2.2) and the public information websites (*M* = 2.1) turn out to be the media sources most accessed.

Considering the interest in receiving additional information regarding the nutritional values of wine, Table 3 shows that participants are very interested (*M* = 4.1). They are also interested in receiving this information through the label (*M* = 4.2). With reference to ingredient list, participants seem less interested than in nutritional information (*M* = 3.5).

With reference to degree of interest in receiving wine nutritional information through alternative sources (beyond labels), Figure 2 reveals that consumers tend to prefer the websites of the manufacturing companies (*M* = 3.50) or public information sites (*M* = 3.34), while the QR code option on the label or Smartphone apps are the least preferred (*M* = 2.85 and *M* = 2.90).

### 4.3. Consumers’ Preferences for Label Formats

Analysing individual bids, Table 4 shows sample’s mean WTP for the four wine labels investigated. The wine for which consumers show higher bids (€4.97) is the wine with the back label with the greatest amount of information content, i.e., the one with the traditional nutritional panel as provided by the Reg. 1196/2011. Furthermore, the D labelled wine—with the indication by means of pictograms of the main nutritional values—obtained a similar WTP (€4.71). In absolute terms, the label which participants express a lower WTP is the one without nutritional information, with reference to the wine in moderation website. It is therefore possible to affirm that participants have expressed a higher preference for the label mode with more informative details, while they attributed less value to the label without nutritional information but only with the indication of the website.

SUR analysis highlights that there are different variables that affect preferences for wine nutritional labels (Table 5). Among socio-demographics variables, gender is the only variable that significantly impacts the WTP for the more informative wine label: women are more willing to pay for more detailed wine nutritional label. Not significant findings were revealed with reference to education or income.

General Health Interest construct (Cronbach’s *α* = 0.82) significantly increases WTP for the most informative wine labels; thus, higher health interest is linked to a higher propensity of respondents to choose the most informative wine label. This suggests that motivational factors also play a central role in influencing nutritional information wants.

Considering the influence of knowledge, our results show that self-reported wine nutritional knowledge significantly affects the WTP for the three nutritional-labelled wines, whereas the (general) nutritional knowledge index affects only preferences for the wine without nutritional information. A possible explanation can be that more acknowledged individuals tend to be less interested to additional information on the topic. Similarly, awareness of linkage between wine and health construct (*α* = 0.65) is significantly related only to the WTP for the wine without nutritional information. In addition, our results reveal that higher wine involvement construct level (*α* = 0.78) decreases preferences for all nutritional labels, in other words product involvement is inversely related to consumers’ information wants.

## 5. Discussion

The debate on the inclusion of nutrition information on labels of wine and spirits has reached a decisive moment as the European Commission is considering the joint self-regulatory proposal of alcoholic beverages industry and in case should evaluate other regulatory as well non-regulatory options. Findings of the current research, while limited in terms of sample representativeness, can offer a useful contribution to the ongoing discussion. According with previous research [15,16,17,18,19], our results show respondents’ low degree of wine nutritional content knowledge and strong support for wine nutritional label. In this regard, as suggested by the seminal paper by Caswell and Padberg (1992), it is important to consider that labelling may act not only as a direct shopping aid but also as a tool of public surveillance assurance [48].

Considering the specific research questions of the current study, results reveal that consumers are interested in nutritional label on wine. Furthermore, results show that the label format most valuable for consumers is the nutritional panel label, which includes all nutritional information, whereas individuals assign the lowest preferences to the less informative wine label (only with a website recall). This second outcome seems contrasting with the joint proposal formulated by the EU alcoholic beverages industry of an online and simplified label, that may be limited to energy value. Indeed, consumers tend to prefer the traditional tool to obtain nutritional information, instead of innovative off-label tools accessible from the label. In addition, recent studies reveal that a relatively small segment of the population is employing those tools as an aid in their purchase decision, especially consumers that are highly involved in wine and seeking deeper information about the product [49]. Furthermore, even if wine sales on the web have witnessed increasing pace, traditional channels are still the most common wine purchasing outlet [50]. Overall, adoption of a new technology into a daily routine takes time, thus information on the product label will remain, at least for a certain time, an important and direct way to deliver accessible information during purchases, allowing easy comparisons among different wine types.

Finally, with reference to our third research question, SUR estimates demonstrate that there are different variables, beyond socio-demographics, that influence consumer preferences for diverse wine nutritional labels. Results confirm that women are more likely to read nutritional labels than men [26] and that women are more prone to seek information while choosing wine [51]. Furthermore, women are also more likely to choose wine based on health evaluations [52]. In addition, and in line with Grunert and colleagues (2018), motivational factors play a central role in influencing nutritional information wants. Higher health interest is linked to higher preferences for more informative wine labels [19].

While in contrast with previous studies, which indicate that information wants regarding the nutritive content of alcoholic drinks are mainly related to the degree of wine involvement [16,19], our outcomes reveal that the latter decreases preferences for wine nutritional labels. This result suggests the existence of different consumer segments with diverse information needs, based on the degree of involvement with the product. More involved consumers, who are very selective in the choice of wine and actively seek for information in their purchasing decisions, could represent the potential market cluster that would most benefit of an e-label, as proposed by the industry.

However, it is important to highlight that the external validity of the current study is limited due the specific shortcomings of consumer laboratory experiments, among others we should underline: the non-random selection of participants and thus non representativeness of the final sample and impossibility to generalise findings, the lack of realism (e.g., lab setting and requesting participants to state their reservation price), the special scrutiny devoted by individuals to the investigated topic, and the obligations of experimental subjects [34].

## 6. Conclusions

WHO considers improving labelling of alcohol beverage as a component of a comprehensive public health strategy to reduce alcohol-related harm. However, in the overwhelming majority of countries worldwide ingredient list and nutritional information are rarely mandatory [3]. Alcohol industry actors are promoting voluntary or self-regulatory initiatives, as alternative to mandatory regimes [53]. In this scenario, the European Commission in 2017 stated that no objective grounds would justify the absence of information on ingredients and nutrition information on alcoholic beverages, and asked the industry for a self-regulatory proposal [7]. The self-regulatory proposal, presented on March 2018, is now under assessment by the EC. To be able to express an opinion, the Commission has to consider conflicting interests. On the one side, the European alcoholic beverage sectors is guided by the intention to respond to consumers’ expectations, while preserving the competitiveness of the operators, as reported in the self-regulation proposal. On the other side, the Commission has to consider consumers associations’ call for more transparency on the labels, in order to inform and educate consumers about what and how much to drink [11,12]. It should also be considered that national legislation of some EU member states is going in the direction of inserting additional nutritional information into the labels of alcoholic beverages (e.g., Austria requires the labelling of the amount of sugar for certain wine products while Romania and Ireland have notified a draft legislation requiring nutrition labelling for particular alcoholic beverages). Therefore, a comprehensive institutional intervention at the European level is desirable to avoid the risk of market fragmentation [10]. Several studies have underlined consumers’ limited knowledge of nutritional value of alcoholic beverage, including wine, and the strong interest for nutritional information [54]. Nevertheless, individual preferences for different formats of information delivery need to be deepened. The present research offers a contribution in this direction. Current results highlight the preference for a traditional and complete form of labelling and indeed support BEUC and EUROCARE objection to off-label information delivery, as proposed by the industry self-regulatory proposal. However, further research should test the robustness of the findings, extending sample size and target, and confronting consumers’ preferences in different countries. In addition, more research is needed on how technology is changing consumers’ information search and purchase decisions, in particular to verify the relative importance of web-link, QR code or other forms of direct access to information on-line.

## Figures and Tables

**Table 1:** Participants’ health interest in food choices, attention towards and use of nutritional labels.

General Health Interest in Food Choices		
I always follow a healthy and balanced diet	Strongly agree	30%
	Totally agree	33%
It is important for me that my daily diet contains a lot of vitamins and minerals	Strongly agree	28%
	Totally agree	37%
It is important for me that my diet is low in fat	Strongly agree	24%
	Totally agree	30%
I am very particular about the healthiness of food I eat	Strongly agree	23%
	Totally agree	37%
I eat what I like and I do not worry much about the healthiness of food	Strongly agree	23%
	Totally agree	10%
**Labelling attention and use**		
I read the nutritional information on the label when I buy food/beverages	Totally agree	45%
I use nutritional information to compare and choose products	Totally agree	42%
I find it difficult to understand the nutritional information on the label	Totally disagree	47%

**Table 2:** Wine consumption variables (%).

Wine consumption frequency	Once a day	15%
	More than once a week	37%
	Once a week	22%
	Less than once a week	9%
	Only on special occasion	17%
Self-reported wine nutritional properties knowledge	None	10%
	Low	36%
	Limited	16%
	Medium	33%
	High	5%
White wine kcal content	Wrong or Don’t know answer	38%
	Correct answer	62%
White wine carbs content	Wrong or Don’t know answer	57%
	Correct answer	42%
Sweet wine kcal content	Wrong or Don’t know answer	69%
	Correct answer	31%
Sweet wine carbs content	Wrong or Don’t know answer	78%
	Correct answer	22%
Have you ever looked for wine nutritional information?	Always	8%
	Seldom	18%
	Rarely	31%
	Once	19%
	Never	24%

**Table 3:** Wine related variables.

Awareness of linkage between wine and health	Mean	S.D.
Excessive consumption of wine could affect my health	4.06	1.10
Compared to other alcoholic drinks wine has more healthy properties	3.78	1.01
A moderate wine consumption could benefit my health	3.57	1.18
Wine consumption contributes to overweight and obesity	3.03	1.13
A moderate wine consumption could help me to prevent some diseases	2.88	1.06
**Wine involvement**		
I have a strong interest in wine	3.21	1.19
I select the wines I purchase very carefully	3.14	1.19
I do not need a special occasion to drink wine	3.60	1.09
I like giving wine as gift	3.40	1.03
**Interest in additional information**		
Interest in additional information on wine nutritional Values	4.12	1.04
Interest in wine nutritional information on label	4.23	1.01
Interest in mandatory ingredient list on wine label	3.54	1.05

**Table 4:** WTP for wine label formats (€).

	Mean	S.D.	Median	Max
**Label A** (kcal per glass)	4.27 ^a^	1.93	4.50	10
**Label B** (Nutritional panel)	4.97 ^b^	1.81	5.00	12
**Label C** (No info only web site link)	3.92 ^c^	2.06	4.00	10
**Label D** (GDA)	4.71 ^d^	1.87	4.50	10

*Note*: Mean WTP with different superscripts (a,b,c,d) are significantly different according to Wilcoxon–Mann–Whitney and Kolmogorov–Smirnov tests (at *p* < 0.00).

**Table 5:** Seemingly unrelated regression (SUR) selected coefficient estimates explaining respondents’ WTP for wine nutritional label.

	Label A (kcal per Glass)	Label B (Nutritional Panel)	Label C (No Info)	Label D (GDA)
Female	0.536	1.187 ***	0.423	0.807 **
Average price paid for a wine bottle (€)	0.094 **	0.063 *	0.127 **	0.085 **
Wine Involvement	−0.807 ***	−0.333 **	−0.777 ***	−0.452 **
General Health Interest	0.255	0.560 **	0.125	0.460 **
Interest in additional information on wine nutritional values	0.712 ***	0.587 ***	0.927 ***	0.793 ***
Self-reported wine nutritional properties knowledge	0.454 **	0.258 **	0.285	0.431 **
Nutritional knowledge Index °	0.715	0.456	1.259 *	−0.031
Interest in mandatory ingredient list on wine	−0.385 **	0.094	−0.534 ***	−0.130
Awareness of linkage between wine and health	0.260	−0.170	0.522 **	0.168
Constant	0.650	0.192	0.713	1.659
*R* ^2^	0.575	0.678	0.533	0.574
*χ* ^2^	139.3	216.8	117.9	139.2
*p*	0.000	0.000	0.000	0.000

*Note*: ***, **, * indicate statistical significance at 1%, 5%, 10% level respectively.° Based on the mean value of items related to objective nutritional knowledge.

## Appendix A

**Table A1:** Internal Consistency of Scale Used.

	Mean Value	SD	Cronbach’s Alfa
Wine involvement	3.32767	0.9367426	0.78
General health interest in food choices	2.47767	0.8050006	0.82
Labelling attention and use	2.097087	0.7819186	0.67
Awareness of linkage between wine and health	3.469903	0.7038876	0.65
